# Noise- and stimulus-dependence of the optimal encoding nonlinearities in a simple ON/OFF retinal circuit model

**DOI:** 10.1186/1471-2202-15-S1-P47

**Published:** 2014-07-21

**Authors:** Braden A W Brinkman, Alison Weber, Fred Rieke, Eric Shea-Brown

**Affiliations:** 1 Department of Applied Mathematics, University of Washington, Seattle, WA 98195, USA; 2 Department of Physiology and Biophysics, University of Washington, Seattle, WA 98195, USA; 3 Program in Neurobiology and Behavior, University of Washington, Seattle, WA 98195, USA; 4 Howard Hughes Medical Institute, University of Washington, Seattle, WA 98195, USA

## 

**Figure 1:**
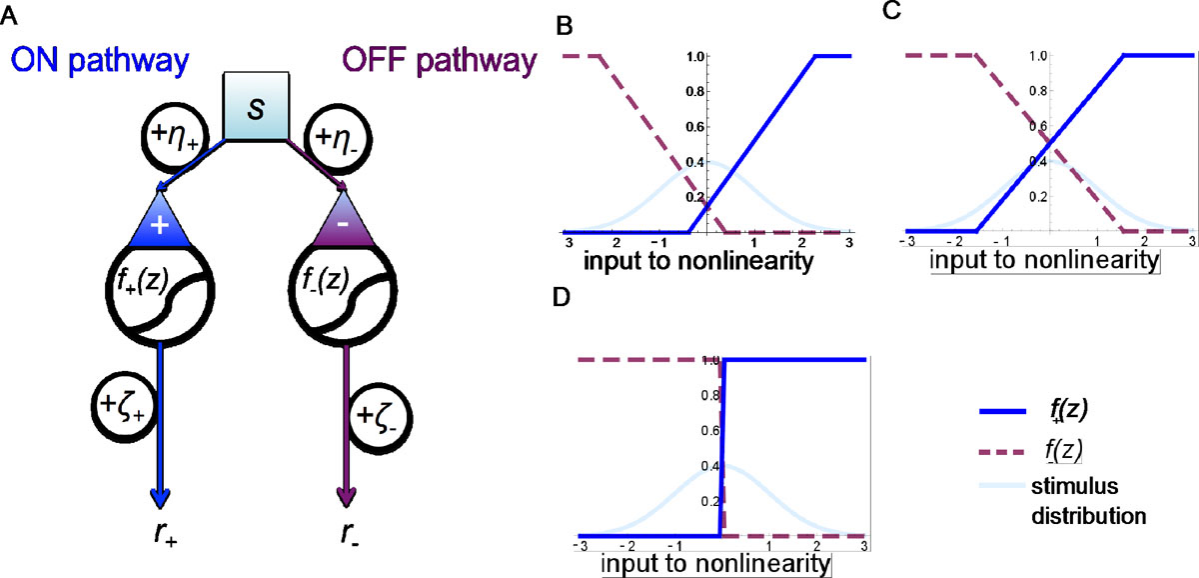
**A.** Simple two-pathway retinal circuit model. A stimulus (*s*) is presented and transmitted to separate ON and OFF pathways, which receive correlated corrupting noises *η*_+_ and *η*_-_, respectively. The signals are passed through encoding nonlinearities to produce inputs *r*_+_ = *f*_+_*(s*+ *η*_+_*)* + *ζ*_+_ and *r*_-_ = *f*_-_*(-s- η_-_)* + *ζ*_-_ to retinal ganglion cells; these responses have been further corrupted by correlated noises *ζ*_+_ and *ζ*_-_. We calculate the optimal shape of the nonlinearities *f*_+_*(z)* and *f_-_(z)* as functions of the noise and stimulus distribution parameters. **B.** The optimal encoding nonlinearities for low pre- and post-nonlinearity noise variance. **C.** Large noise variances. **D.** Very large post-nonlinearity noise.

Encoding of stimuli in the retina depends on the statistical properties of the input stimuli, neural noise, and circuit nonlinearities. Here, we present a simple model of a two-path ON/OFF RGC circuit (figure 1A). We use variational methods to analytically calculate the optimal encoding nonlinearities in the presence of noise sources with two key biophysical properties: they have separate components that corrupt the stimulus (pre-nonlinearity) and the responses (post-nonlinearity), and they may be correlated across cells. We study qualitatively the effects of the competition between the stimulus and noise sources on the form of the encoding nonlinearities. We find that when both pre- and post-nonlinearity noises are low, the ON and OFF pathways each encode roughly half of the stimulus distribution (figure 1B). However, the optimal nonlinearities rearrange at higher noise levels, introducing redundancy in signal encoding (figure 1C). For very large post-nonlinearity noise, the best the circuit can do is encode the sign of the received stimulus (figure 1D). The results of related studies are consistent with behavior observed in specific parameter regimes of the broad framework encompassed by this model [1,2].
